# A Qualitative Study of Patients' Beliefs and Perception on Medicinal Properties of Natural Hot Spring Bath for Musculoskeletal Problems

**DOI:** 10.1155/2020/3694627

**Published:** 2020-07-09

**Authors:** Binit Vaidya, Shweta Nakarmi

**Affiliations:** National Center for Rheumatic Diseases, Kathmandu, Nepal

## Abstract

Natural therapy modalities such as thermal therapy and balneotherapy are commonly being practiced for the management of chronic aches and pain all over the world. Nepal has many such natural hot water springs among which few are famous for therapeutic purposes. Thousands of people with some musculoskeletal problem visit those places in the hope of getting rid of their problems. This study aimed to understand their belief in such therapies, expectations, and satisfaction after treatment along with their knowledge of the safety of hot spring water bath. Among 126 participants interviewed, 31% had inflammatory arthritis, followed by degenerative disorders in 29.4% and soft-tissue rheumatism in 12.7%. Around three-quarters believed that hot spring water has natural healing power and thus can improve their pain. Many even believed that water in natural springs is devoid of any chemicals. So, it is a safe treatment option. Regarding the expectation of cure, they had mixed opinions. Naïve participants hoped they might find a permanent cure in thermal baths. However, repeated visitors said that the effect usually lasted for a few months and they have to visit there regularly. Almost two-thirds of people thought that such natural treatment does not have any side effects. Few stated that they had faced certain problems after the treatment. The water tested from the study site showed that it contained a higher amount of chlorine and sulfate in comparison to other hot water springs in Nepal. The minerals present in water might be a cause of temporary relief of pain. Also, outbreaks of infection from common spring baths have to be considered as such cases have been reported in the past. In conclusion, the medicinal benefits of such natural hot water springs have to be studied further and awareness regarding safety should be given to the people seeking treatment.

## 1. Introduction

Musculoskeletal pain is very common in the general population affecting around 20% of the adults [1]. People of all ages complain of musculoskeletal pain due to various reasons at some time during their life. The causes may be mechanical, degenerative, inflammatory, and so on [1, 2]. Most of the inflammatory and degenerative problems persist for a long duration and people tend to seek various medical options for themselves. In addition to allopathic treatment, various alternative medicines and the natural therapies are sought by these patients [3]—one of them being a visit to natural hot spring pools [4–6].

In Nepal, though there are 23 natural hot springs [7], the one located at an altitude of 2474 meters above sea level at the Myagdi district is famous amongst people with musculoskeletal pains for its medicinal properties. A few environmental studies have shown that these hot springs contain an abundance of dissolved solutes including sodium bicarbonate, chloride, and sulfur [6–8] which might have beneficial effects on skin diseases [9, 10] and some benefit on arthritis symptoms [11]. It is well known that heat therapy and hydrotherapy are effective modes of pain alleviation in cases of arthritis [12]. However, natural hot springs are a habitat for many free-living organisms which might be a potential health hazard, not to mention the risks of sharing a pool [13, 14].

The Myagdi natural hot water spring being very popular amongst patients with rheumatic diseases, we planned this survey to examine the understanding, attitudes, beliefs, and perceptions of these patients and address these factors to promote further research in natural healing properties of such spring and to systematize the services provided.

## 2. Methods

### 2.1. Study Setting and Participants

The study was conducted at a religious natural hot spring source located in the central west region of Nepal. The spring is located at an elevation of 2474 meters from the sea level and is frequented by around 25,000 visitors in a year for its medicinal properties. The potential participants stay within the premises for a period varying from few days to few months for the treatment. Before initiation of the study, the documents and procedures related to the study were approved by the institutional review board of the National Center for Rheumatic Diseases (NCRD), Kathmandu, Nepal, and were compliant according to The Belmont Report [15]. A prior notification about the study was given to the participants through the local community leader and via written handouts. An informed but verbal consent was obtained from all. All potential participants were related to some kind of musculoskeletal problems.

### 2.2. Observer/Interviewer

The principal investigator was a rheumatologist working at NCRD. The other two interviewers were postgraduate doctors in internal medicine trained in the evaluation and treatment of rheumatic diseases.

### 2.3. Tools

A semistructured questionnaire was used to interview the participants to understand their beliefs and perception regarding the effectiveness of natural hot water spring in relieving musculoskeletal problems. They were guided by focused questions to express the reason for their beliefs, their attitude, behavior, and motivation to seek benefits of such natural hot spring. The questions were also designed to assess the fulfillment of their expectation after seeking treatment.

### 2.4. Procedure

A field observation was conducted among the participants. Purposive sampling was used where the participants were identified by prior notification of the study purpose to all the attendees of the hot spring and selecting those who were seeking medical care for their musculoskeletal symptoms. The sample size in field observation was determined by data saturation according to the grounded theory [16]. Each participant who agreed to participate was taken to an interview hall next to the hot spring pool and was offered free medical screening for rheumatic diseases as an incentive.

### 2.5. Data Collection and Analysis

Participant interview was conducted in the local Nepali language. A semistructured questionnaire helped to focus the interview on patients' beliefs, attitudes, and outcomes regarding the use of natural hot spring baths for curing their problems. Participants were also interviewed about their perception of long-term medicinal benefits with the use of hot springs. Each interview lasted between 10 and 15 minutes and main features were collected as written transcripts by the interviewer. An analytic induction was used to develop hypotheses and analytical categories, and they were further tested in other participants. Limited demographic data was obtained.

## 3. Results

A total of 126 participants were interviewed by 3 observers. All were above 18 years of age with a mean age of 52.8 ± 14.9 years (19–86 years) and were mentally sound. Of the participants, 72.2% were females. The most common musculoskeletal problem was inflammatory arthritis (31.0%), followed by degenerative disorders (29.4%) and soft-tissue rheumatism (12.7%).

Analysis of interview transcripts revealed three main analytical categories as follows:

- Perception and Acceptance of Hot Spring

More than three-fourths of the respondents perceived that the natural hot spring has natural healing powers. A variety of reasons were offered. Some believed that the natural source of water did not have any *chemical contamination* as illustrated by following statement: “nature has the potential to cure the ailments and would be better than any chemical drugs”; “no chemicals and drugs are involved in treating my pain, and I believe this is the best part.”

Some participants, who inhabited nearby villages, commented that as they are geographically benefited compared to people who come from a distance, they would prefer to use the “*blessing*” they received from nature: “It is easy for me. I just dip in the spring for few days whenever I feel aches and pains”; “the pool has divine powers and has been worshiped by our ancestors.”

The cost was another factor for the acceptance: “If they were commercial, they would not provide the stay free”; “They charge 50 Nepalese rupees per person as maintenance fee and I think this is a service motive.”

(b) *The Need for Perseverance and Expectation of Cure*

Participants who were visiting the hot spring for the first time were very hopeful that their ailments could be *cured*: “I have tried many options but by [*sic*] pains keep coming. I came here with a belief that spending some time in this pool can cure me permanently”; “I have met many people who were cured after coming here.” However, few local inhabitants and repeated visitors believed that the pains do improve to some extent but need *repeated visits*: “I usually come here every year before or during winter. I feel better after a week of daily dipping in this spring, but the pain comes back every year”; “Anyways you need medicine for life. It is better that I keep coming here once or twice a year”; “I am not sure if it can cure my arthritis, but what is the harm in trying?”

The next two groups were induced during the initial few interviews and were further probed in subsequent interviews:

(c) *Awareness of Side Effects*

More than two-thirds of participants believed that there is no side effect in seeking natural treatment. As understood in the prior section, many believe that the water is free of chemicals that drugs have and thus would be safer than medicines. “This is my fifth visit here and I usually stay for 7 to 10 days in the pool. Sometimes, I even drink this water when I have worse pain. But I have *never experienced any side-effects*”; “There are hundreds of people here and I have never heard anyone talking of side-effects.” However, a minority of respondents reported intolerance: “My pain gets better but my urine stops and burns whenever I try this pool,”a lady said. “My swellings increased in 3^rd^ day of dipping, I do not know whether I should continue,” a person who was later diagnosed as scleroderma by the team rheumatologist complained.

(d) *Peer-Pressure*

Around 30% of participants and most who were visiting for the first time narrated the importance of pressure from peers and relatives in visiting. Most of the participants emphasizing the importance of peer pressure were females. The following statements highlight this: “I was better with medications too but my mother wanted me to visit here. It seems she was cured here long back”; “I do not believe, neither do I refuse that this spring cures the pains, but I did not want to say no to my family.” One participant said “I was not aware that we came here for medicinal purposes. I came with my family for an outing and was taken by surprise. However, no harm was done!”

When asked about the issues they would like to mention in addition to the focused questions, the most common responses were regarding the *privacy and cleanliness* of the pool. Being used by hundreds of people in a day, they were not sure if the sanitation is maintained: “It looks unhygienic but maybe the natural property of hot water inhibits pathogens” was a popular belief. Also, though time slots were different for male and female visitors, the open space provided little privacy: “There are no proper and adequate changing rooms but everyone is doing it, so I felt less awkward,” a lady mentioned. Also, few elderly people mentioned the need for medical facility in case any emergency occurred: “I am a heart patient. If anything happens to be [*sic*] with hot water I do not know what to do!” explains the bewilderment of that group of participants.

## 4. Discussion

Musculoskeletal pain is one of the common complaints in Nepalese people [17], maybe because of its hilly landscapes, culture, and lifestyle. Rheumatology is still a developing subject in Nepal with very few people being aware of rheumatic diseases and their treatment modalities. Many patients seek alternative modalities of treatment including natural therapies [3]. One of the most popular natural therapy options often pursued by them is the natural hot spring pool located in the central west region of Nepal. The location was chosen based upon the feedback and beliefs of many patients attending a tertiary care rheumatology center in Kathmandu where the primary investigator is affiliated.

Though there have been no prior studies on the patient perception about such natural therapies in Nepal, overall a few pieces of research are showing the effectiveness of hot water therapy [4, 5], balneotherapy, and sulfur bath therapy [18] for patients with various kinds of arthritis. Sulfur has been used as a complementary therapy for its anti-inflammatory properties [8, 11]. A study back in 1990 had divided rheumatoid arthritis patients into four groups receiving heated mud, a sulfur bath, combination, and control groups and showed that all the three treatment groups had beneficial effects up to 3 months as compared to the control group [18]. Few studies in patients with osteoarthritis of hand and knee showed a beneficial effect of the sulfurous thermal bath as compared to normal hot water bath [19, 20]. Studies in patients with hip and knee osteoarthritis have also shown a beneficial effect of treatment with sulfur or mineral containing bath as compared to exercise alone [21]. These observations indicate a scientific basis for this healthcare-seeking behavior of musculoskeletal pain patients in our set-up. However, their perception that there are no chemicals involved was not appropriate as the water tested had many dissolved solutes including chloride and sulfur. Studies in the past have shown that these natural hot water springs contain certain minerals which may be beneficial for chronic inflammatory conditions. A report by Ranjit M in the early 90s showed that the majority of these thermal springs contain a high quantity of chlorine, sulfur, bicarbonate, carbon dioxide, and calcium [7]. Water in springs of Tatopani, our study location, is considered mature with relatively high chloride (255.5 mg/L) and sulfur (217 mg/L) content as shown in Table 1. Chloride content is higher in comparison to other natural hot spring water like Tapoban (50.1 mg/L), Jomsom (95.9 mg/L), and Rasuwa (0 mg/L). Similarly, the quantity of sulfate is also higher in comparison to Tapoban (25.9 mg/L) and Rasuwa (0 mg/L) [7].

The expectation for the cure of the ailments was observed mainly in naïve visitors, whereas the old ones accept the temporary nature of the benefit. It can be hypothesized that the local efficacy usually disappears after a while. Separate analysis was not done to observe the effect of religious belief on the benefits obtained from the hot spring. Those cases with persistent nature of arthritis are bound to have recurrence once the beneficial effect of any form of treatment wanes. However, we felt the need of spreading awareness among them to identify the alarming features of damaging and deforming disease who are bound to receive disease-modifying drugs with or without such natural therapies, as exemplified by detecting one previously undiagnosed scleroderma patient among them.

Most of the visitors spent an average of 7 days, maximum up to a month at the hot spring. Many of them were repeat visitors. The finding that the majority of them believed that this process of seeking medical benefits was devoid of side effects was unexpected. Natural hot springs are known habitat for free-living amoeba which can be rapidly lethal pathogens [13, 14, 22, 23]. Though the rare, serious amoebic infection has been reported in Kathmandu, Nepal, too, a fatal case due to free-living amoeba *Naegleria fowleri* has been reported from Kathmandu valley recently [24]. Studies from other Asian countries have reported frequent isolation of thermophilic *Acanthamoeba* [13, 14] and *Naegleria* species [22, 23] in therapeutic hot springs. Japanese authors have reported an outbreak of *Legionella* pneumonia with 295 documented cases and 7 deaths in a hot spring bath within 1 month. They attributed it to the following causes: insufficient knowledge and understanding of stuff on *Legionella* and legionellosis; (2) residual water in tubing system after trial runs which might lead to multiplication of legionellae in it; and (3) inadequate disinfection and washing for whole circulation system before the experience bathing [22]. We can easily derive the fact that the hot spring location studied in Nepal also has similar risk factors and a need for awareness of the authorities cannot be overemphasized.

We believe that while the medicinal properties of natural hot spring baths need to be explored further scientifically and utilized with a public health benefit, a proper method for creating awareness of the disease and possible risks associated must be addressed to timely prevent the unwanted disasters.

## 5. Conclusions

Thermal water bath therapies are beneficial for the alleviation of chronic pain. Various minerals present in natural hot spring pools provide added benefits. However, it can only be used as additional therapy for patients with rheumatic diseases along with specific treatments. Hazards like infection should also be taken into consideration.

## Figures and Tables

**Table 1:** Chemical analysis results for water from the study site in comparison to other natural hot spring waters.

SN	Parameters	Test results from the study site (Myagdi)
1	Temperature (°°C)	23
2	pH	6.95
3	Conductivity (*μ*s/cm)	1902
4	Turbidity (NTU)	0.76
5	Total hardness (mg/L as CaCO_3_)	190
6	Chloride content (mg/L)	255.6
7	Iron content (mg/L)	0.1
8	Arsenic content (mg/L)	ND
9	Ammonia (mg/L)	1
10	Nitrate (mg/L)	ND
11	Sulfate (mg/L)	217
12	Total coliform count (count/100 ml)	0

## Data Availability

The data used to support the findings of this study are available from the corresponding author upon request.
